# Ionic Conductivity Enhancement in UHMW PEO Gel Electrolytes Based on Room-Temperature Ionic Liquids and Deep Eutectic Solvents

**DOI:** 10.1021/acsapm.2c00104

**Published:** 2022-03-25

**Authors:** Víctor Gregorio, Nuria García, Pilar Tiemblo

**Affiliations:** Instituto de Ciencia y Tecnología de Polímeros, ICTP-CSIC, Juan de la Cierva 3, 28006 Madrid, Spain

**Keywords:** polymer gel electrolytes, Li, deep eutectic solvents, ionic liquids, UHMW PEO, self-healing, energy storage, batteries, ionogel

## Abstract

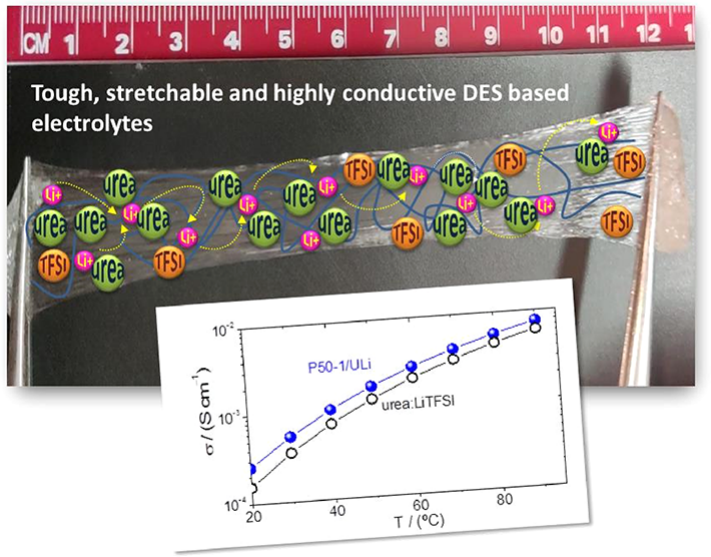

Physical gels made of poly(ethylene oxide) (PEO) and deep eutectic solvents urea-Li bis(trifluoromethanesulfonyl)imide (TFSI) and ethylene glycol/LiTFSI, or pyrrolidinium ionic liquid solutions PYR13TFSI-LiTFSI and PYR14TFSI-LiTFSI, are prepared by a fast, single-step process, which involves no auxiliary solvents or intermediates and is reproducible and scalable. The properties of these gels are studied as a function of the PEO content and its molecular weight and the nature of the liquid electrolyte. The gels prepared with a low concentration (1–5 wt %) of ultrahigh molecular weight (UHMW) PEO are tough, stretchable materials which resemble soft elastomers and are also self-healing and transparent. Their rheology shows the conventional behavior of physical polymer gels, so that the higher the molecular weight of PEO, the lower the polymer concentration needed to produce the gel. However, the ion conductivities and diffusivities of the gels are striking, in many cases being equal to or significantly higher than those of pure liquid electrolytes. This ion conductivity enhancement is the highest for the lowest PEO concentration with the highest molecular weight. This unprecedented molecular weight dependence of conductivity and diffusivity is the result of two combined effects: the liquid electrolyte chemical structure modification as a consequence of the addition of PEO and the development of elastic networks, where ion mobility and rheology are uncoupled when the PEO added is of UHMW.

## Introduction

The search for solid-like electrolytes for metal batteries, either for the well-known Li-based or the newer Na, Zn, Ca, or Mg metal-based ones, is a fascinating topic of much technological projection. Solid electrolytes are the path toward safer batteries, not only because they prevent the leakage of liquid electrolytes and are often toxic but also because they mitigate or even avoid dendrite growth and the subsequent short-circuits.^1^ Among solid or quasisolid electrolytes, those based on polymers, either solid-like or gels, have many advantages. At their best, they can be light, flexible, and tough. They allow for good adhesion with the electrodes at the interfaces, and they can be industrially produced by simple processing methodologies that are sustainable and scalable. In addition, in order to have safer batteries and more simple recycling processes, flammable organic solvents such as cyclic carbonates must be avoided. For that purpose, some years ago, the use of non-flammable room-temperature ionic liquids (RTIL) began to be explored as electrolytes, even if their high viscosity makes their ionic conductivity lower than those of cyclic carbonates. However, most ionic liquids have the drawback of being expensive. Because of that, the focus has more recently been on a specific type of ionic liquid, namely, deep eutectic solvents (DESs);^2^ more specifically, those made out of inexpensive and available compounds.

The first research articles on polymer gel electrolytes of DES date from the early 2010s and refer to the preparation by solvent casting of a Li gel electrolyte based on corn starch, using the well-known urea-choline chloride DES and LiTFSI as the Li salt.^3^ In the literature, other methodologies to obtain solid or gel electrolytes from DES relying on the use of polymers can be found, such as poly(vinyl alcohol) electrospun membranes soaked with *N*,*N*-diethylethanolammonium chloride and ethylene glycol (EG) DES,^4^ in situ monomer polymerization,^5,6^ or in situ polymer cross-linking.^7^ Moreover, it is possible to obtain gels without the addition of polymers, for instance, by gelation of *N*-methyl acetamide-LiTFSI with tetraethylorthosilicate and formic acid,^8^ or self-assembled gels formed by ionic interactions of 1,3:2,4-dibenzylidene-d-sorbitol in different DESs based on choline chloride.^9^ In any case, whenever polymers are used as gelling agents, the preparation procedures found in the literature involve either the use of auxiliary solvents, which need to be evaporated, and/or chemically cross-linked gel electrolytes, which are impossible to reshape and difficult to recycle.

The absence of auxiliary solvents and a thermosensitive rheology are very recommendable characteristics in electrolytes, which will eventually require upscaling. From the perspective of a polymer scientist, turning a liquid into a gel or a plasticized solid can be done by using ultrahigh–molecular-weight (UHMW) polymers to produce physical gels. Physical gels have the added advantage over chemical ones of being processable as thermoplastic polymers because their rheology is temperature-sensitive. Correctly chosen UHMW polymers can be soluble in the liquid DES electrolyte without the need of any auxiliary solvent. In previous work on gel electrolytes for Al secondary batteries,^10^ this strategy has been used to prepare gel electrolytes with the DES urea/AlCl_3_ and UHMW poly(ethylene oxide) (PEO). It has been demonstrated that above a given molecular weight (>9 × 10^5^ g mol^–1^), the entanglements of the long PEO chains succeed to produce gels with the DES urea/AlCl_3_ with very low polymer concentrations. Moreover, the higher the molecular weight of PEO, the lower the concentration required to obtain a gel. Tough and stretchable gels can be obtained in this way, which are very sticky and wet the electrodes well. The preparation procedure is simple, consisting of stirring the powdery PEO in the DES urea/AlCl_3_ while increasing the mixing temperature over 65 °C, at which the PEO melts.

The use of UHMW polymers to prepare gel electrolytes has other very interesting features. In 2014, Archer and collaborators reported for the first time a low-modulus cross-linked polymer gel electrolyte, where dendrite suppression was observed.^11^ In the following years, they reported on the electrochemical performance of UHMW PMMA and organic liquid electrolytes (propylene, ethylene carbonate, and dimethylsulfoxide) containing Li, Na, Zn, and Cu salts.^12^ The authors observe how, under a polymer concentration threshold, the ionic conductivity of a liquid is negligibly affected, whereas the electrolyte becomes viscoelastic.^13^ In Archer and collaborators’ work,^12,13^ the polymer role is to reduce the electroconvection during the deposition of the metallic cation at the anode. Therefore, care is taken to choose those polymers that interact with the liquid electrolyte as little as possible. The reduction of electroconvection yields homogeneous metal deposits with no dendritic growth. This is extremely interesting because the general approach to reducing dendrites involved a mechanical resistance to their growth,^14^ a strategy that requires polymer electrolytes with a relatively high elastic modulus and consequently low ion mobility. In contrast, the strategy proposed by Archer and collaborators can produce gels that both mitigate or eliminate dendritic growth and preserve the ionic conductivity of a liquid. This strategy can make polymer gel electrolytes a real option for secondary batteries based on Li or other metallic cations such as Na, Zn, or Al.

Following this very appealing path, in this work, ion gels of PEO of different molecular weights and concentrations, DES urea/LiTFSI, and EG/LiTFSI are prepared using a fast and scalable procedure previously reported by us for urea/AlCl_3_ PEO gels.^10^ Notably, in those gels, no dendritic growth was detected when studying Al deposition at the anode.^15^ The eutectic mixtures chosen, urea/LiTFSI and EG/LiTFSI, are simple to prepare and contain inexpensive, benign, and accessible components such as urea or EG. The eutectic mixture urea/LiTFSI, first described in 2001,^16^ is very interesting because of its reasonably wide electrochemical window (over 4 V) and its high Li transport number.^17,18^ On its turn, EG/LiTFSI has been recently described^19^ as a hazardless and cheap DES with superior electrochemical properties: high electrochemical window (∼4.5 V vs Li^+^/Li), high thermal stability (∼200 °C), good flame resistance, as well as a relatively high ionic conductivity of about 3 × 10^–3^ S cm^–1^ at 30 °C. To further prove the validity of the approach to other electrolytes, Li-doped pyrrolidinium ionic liquids are also included in this study. Whereas Archer and collaborators^12,13^ chose polymers that would not interact with liquid electrolytes, in this work, interaction is sought between PEO and liquid electrolytes. Some of the PEO ionogels presented in this work display a significant enhancement of ion mobility, which depends on the PEO molecular weight and concentration; this unprecedented enhancement of ion mobility in polymer gel electrolytes is characterized and studied comparatively to the polymer electrolytes’ rheology.

## Experimental Section

### Materials

LiTFSI was received from Sigma-Aldrich (MO, USA) and dried under vacuum for 24 h at 100 °C before use. Urea from Sigma-Aldrich and EG from Alfa Aesar (Thermo Fisher, Kandel, Germany) were used as hydrogen bond donors (HBDs). Ionic liquids 1-propyl-1-methylpyrrolidinium bis(trifluoromethylsulfonyl)imide (PYR13-TFSI) and 1-butyl-1-methylpyrrolidinium bis(trifluoromethylsulfonyl)imide (PYR14-TFSI) are from Solvionic, Toulouse, France. Once opened, the liquids were kept in desiccators. PEO with molecular weights, *M*_w_ = 1 × 10^5^, 9 × 10^5^, 20 × 10^5^, 50 × 10^5^, and 80 × 10^5^ g mol^–1^, from Sigma-Aldrich, were used to prepare the gel electrolytes. Prior to being used, PEO was kept under vacuum, and urea and LiTFSI were kept at 100 °C overnight.

### Polymer Gel Preparation

Urea/LiTFSI and EG/LiTFSI have been prepared as described elsewhere.^16,20^ Briefly, the DES are prepared by stirring LiTFSI and a hydrogen bond acceptor (HBA) with urea or EG at 80 °C until a transparent homogeneous liquid is formed. The nomenclature and composition of the ionic liquids are shown in Table 1, including the HBD/HBA molar ratio and the HBA molar fraction, *f*, in the last column of the table. The HBD/HBA molar ratios have been chosen in each case as the ones producing the DES with the highest ionic conductivities.^16,19^ Gels have also been prepared with LiTFSI-doped PYR13-TFSI and PYR14-TFSI, for the sake of comparison with our previous results^21−23^ and with the general literature. The concentrations employed are shown in Table 1.

**Table 1:** Nomenclature and Composition of the DES ULi 3.5:1 and EGLi 4:1, and of the LiTFSI Dissolutions in PYR13-TFSI (Pyr13Li) and PYR14-TFSI (Pyr14Li)

DESs							
		HBD			HBA		
	HBD/HBA mol ratio	HBD	wt %	mol L^–1^	wt %	mol L^–1^	*f*
ULi	3.5:1	urea	43	9.52	57	2.64	0.22
EGLi	4.0:1	EG	46	9.06	54	2.30	0.20

RTIL solutions							
		solvent			salt		
	RTIL/salt mol ratio		wt %	mol L^–1^	wt %	mol L^–1^	*f*
Pyr13Li1	4.0:1	PYR13TFSI	85	2.96	15	0.74	0.20
Pyr13Li2	2.0:1	PYR13TFSI	74	2.56	26	1.28	0.33
Pyr13Li3	1.0:1	PYR13TFSI	60	2.05	40	1.95	0.49
Pyr14Li2	2.0:1	PYR14TFSI	75	2.51	25	1.23	0.33

**Scheme 1:**
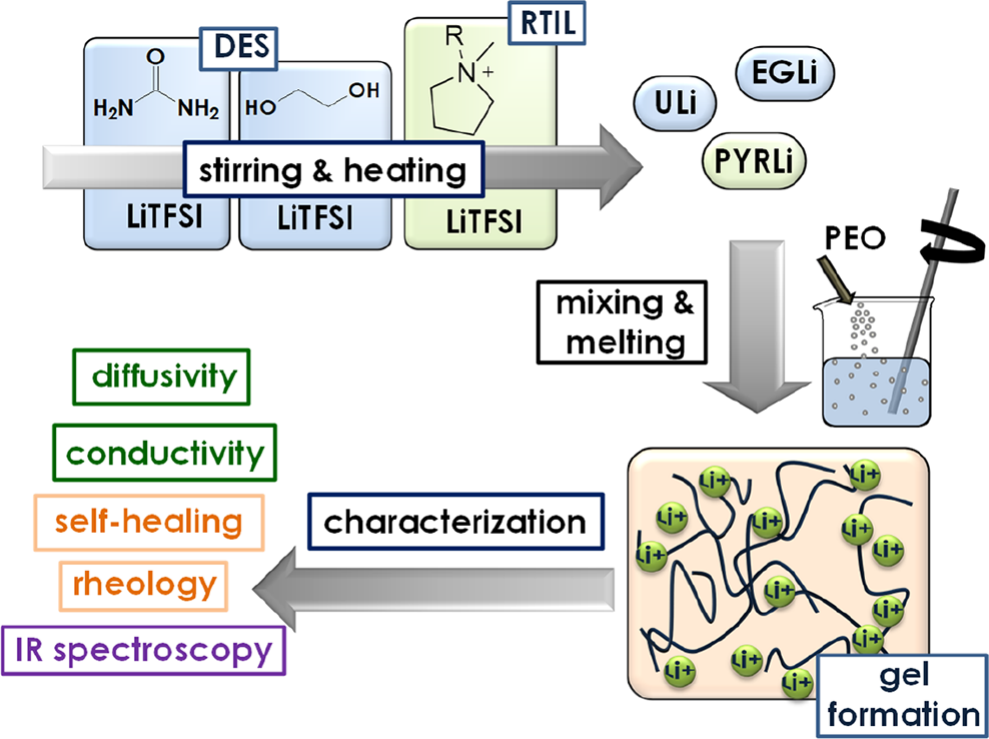
Preparation and Characterization of the PEO Gel Electrolytes

Gel electrolytes were prepared by adding 1, 2.5, 5, and 7.5 wt % of the powdered polymer to the liquid electrolyte at room temperature and mixing it manually while increasing *T* to 70 °C, above the melting point of PEO. The mixture is then stirred constantly at 70 °C for another 10 min, as described for aluminum electrolytes.^10^ This methodology, illustrated in Scheme 1, is simple and renders very reproducible samples. Moreover, it does not involve the use of solvents. In a previous work,^23^ we showed that this manual mixing method yields very reproducible rheological and electrochemical results when compared to industrially scalable melt compounding procedures such as extrusion.

The gels are named as Pm-*n*/X, where m stands for PEO MW × 10^–5^ g mol^–1^, *n* stands for the PEO concentration in wt %, and X stands for the DES employed, ULi for urea/LiTFSI or EGLi for EG/LiTFSI, or RTIL solutions with different LiTFSI concentrations, Pyr13Li1, Pyr13Li2, and Pyr13Li3 for Pyr13TFSI-LiTFSI, and Pyr14Li2 for PYR14TFSI-LiTFSI.

### Characterization

The rheological measurements of the gel electrolytes were performed using an AR-G2 rheometer with a 25 mm diameter stainless parallel plate steel geometry in a frequency range of 0.01–100 rad s^–1^ for the gels with 5 wt % of PEO and a range of 0.06–100 rad s^–1^ for the gels with 1 wt % of PEO. Measurements were carried out at 25 and 75 °C prior conditioning for 15 min at a temperature of the experiment.

The ionic conductivity (σ) of the electrolytes was measured using a dielectric spectrometer (DETA NOVOCONTROL GmbH Concept with a high-performance frequency analyzer Alpha-A in combination with a QUATRO Cryosystem) from −50 to 90 °C in the frequency range of 1–10^7^ Hz. Electrolytes were placed between two stainless steel electrodes with a diameter of 10 mm covered by a Teflon scaffold of 700 μm thickness to avoid creep during measurement. The electrolyte is cooled down to −50 °C, and measurements are registered every 10 °C up to 90 °C. Then, the electrolyte is cooled down, measuring from 85 to 25 °C every 10 °C. This protocol ensures that any effect of phase transitions or relaxations on σ can be detected, providing information on the state of the sample and the reproducibility of the result. The data at 25 °C in Table 2 correspond to the measurements carried out in the cooling scan. The data on cooling, though very similar, are slightly higher than those obtained on heating, as shown for the electrolytes Pyr14Li2 and P50-1/Pyr14Li2 in Figure S1. Scattering of the values of σ measured on different batches of the same formulation remains below ±15%.

**Table 2:** Composition of the ULi, EGLi, Pyr13Li, and Pyr14Li Gels Prepared with PEO of Different MWs and Their Li^+^/EO Molar Ratio and Rheology at 25 and 75 °C Characterized by the Crossover Frequency, *G*′ = *G*″, the Elastic Modulus at 100 rad s^–1^, *G*′_100_, and Ionic Conductivity (σ) at 25 °C

				rheology			
	PEO			25 °C	75 °C		σ × 10^3^ S cm^–1^
electrolyte	MW × 10^–5^ g mol^–1^	wt %	Li^+^/EO molar	*G*′ = *G*″, rad s^–1^	*G*′ = *G*″, rad s^–1^	*G*′_100_, Pa	25 °C
**ULi**		0					0.29
P1-1/ULi	1	1	8.65	>100	>100		0.29
P9-1/ULi	9	1	8.65	26.7	38.5	100	0.32
P20-1/ULi	20	1	8.65	0.87	17.0	100	0.36
P50-1/ULi	50	1	8.65	<0.01	0.30	100	0.40
P80-1/ULi	80	1	8.65	<0.01	0.08	100	0.41
P50-2.5/ULi	50	2.5	3.41	<0.01	<0.01	500	0.30
P1-5/ULi	1	5	1.66	>100	>100		0.33
P9-5/ULi	9	5	1.66	0.06	1.00	2000	0.30
P20-5/ULi	20	5	1.66	0.03	0.20	2500	0.27
P50-5/ULi	50	5	1.66	<0.01	<0.01	3100	0.22
**EGLi**		0	0.25				2.70
P1-1/EGLi	1	1	8.20	>100	>100		2.50
P50-1/EGLi	50	1	8.20	0.11	0.40	50	3.10
P1-5/EGLi	1	5	1.57	>100	>100		1.92
**Pyr14Li2**		0					0.37
P1-1/Pyr14Li2	1	1	3.79		>100		0.47
P50-1/Pyr14Li2	50	1	3.79		0.20	200	0.69
P1-5/Pyr14Li2	1	5	0.73		>100		0.53
P50-5/Pyr14Li2	50	5	0.73		<0.01	1300	0.81
**Pyr13Li1**		0					1.60
P50-1/Pyr13Li1	50	1	2.28		31.4	50	1.58
P50-5/Pyr13Li1	50	5	0.44		0.046	700	1.17
**Pyr13Li2**		0					0.76
P50-1/Pyr13Li2	50	1	3.95		3.60	100	1.58
P1-5/Pyr13Li2	1	5	0.76		>100		0.86
P50-5/Pyr13Li2	50	5	0.76		<0.01	1300	1.17
P50-7.5/Pyr13Li2	50	7.5	0.49		<0.01	2600	0.93

The diffusion coefficients for Li and TFSI ions, *D*_Li_ and *D*_TFSI_, respectively, were determined by pulsed gradient solid state (PGSE) solid NMR. For the ^7^Li and ^19^F PFG NMR measurements, portions of the gels were placed in a 5 mm o.d. NMR tube. The measurements were performed using a Bruker AVANCE Neo 400 spectrometer (Bruker BioSpin GmbH, Rheinstetten, Germany) equipped with a 89 mm wide bore and a 9.4 T superconducting magnet (Larmor frequencies of ^7^Li and ^19^F at 155.51 and 376.51 MHz, respectively). The ^7^Li and ^19^F diffusion data were acquired at 25 ± 0.1 °C with a Bruker diffusion probe head, Diff50,using a simulated spin-echo pulse sequence. Typical 90° radiofrequency (rf) pulse lengths varied between 10 and 12.0 μs, and the diffusion time was fixed at 40 ms in all experiments. Scattering of diffusion coefficients is lower than ±15%.

Self-healing experiments were carried out by cutting through the gel electrolyte (several mm) inside a vial and recording the evolution of the gel along time with digital images. The vials were placed upside down during the whole experiment to reduce the creeping effect.

Additional elongation and self-healing tests were done by stretching the gel fixed with tweezers at both sides up to the break point. Then, both parts are put together and successively stretched.

The FTIR spectra were recorded in ATR mode using an FTIR Perkin-Elmer Spectrum-One (PerkinElmer, Waltham, MA, USA), with four scans and a resolution of 4 cm^–1^.

## Results and Discussion

Table 2 collects all the blends prepared for this work. The gels’ nomenclature is explained in the Experimental Section. As an example, P50-1/ULi is a gel prepared with the DES ULi and 1 wt % of PEO 50 × 10^5^ g mol^–1^. Table 2 includes ten blends of ULi and PEO, varying the PEO MW from 1 × 10^5^ to 80 × 10^5^ g mol^–1^ and its concentration from 1 to 5 wt %. With EGLi, Pyr14Li, and Pyr13Li, blends have been prepared at 1 and 5 wt % using the PEO 1 and 50 × 10^5^ g mol^–1^.

In DES electrolytes, it is not possible to substantially modify the concentration of the components, which is roughly fixed by the eutectic point composition. To study the effect of the Li concentration on the formation and properties of these gels, it is necessary to employ salt dissolutions, and not eutectic mixtures. For this purpose, Li-doped TFSI ionic liquids,^24^ well-known for their excellent electrochemical properties, have been used. In this way, a very complete survey of the effect of polymer concentrations, MW, and nature of the electrolyte on rheology and ionic conductivity can be performed.

**Figure 1:**
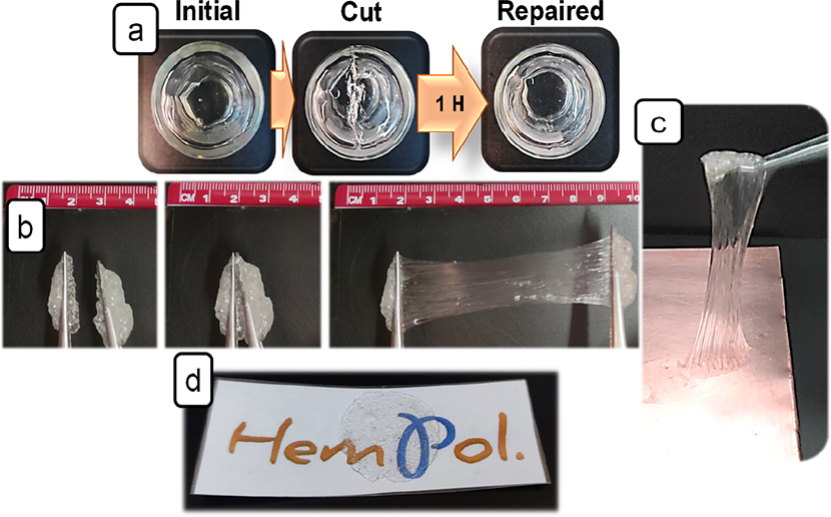
Pictures showing physical features shared by all the ionogels in this work: self-healing ability of P50-5/ULi (a), stretching immediately after sticking back two portions of P50-5/EGLi (b), adhesion properties of P50-5/ULi on a copper foil (c), and transparency of P50-7.5/Pyr13Li2 (d).

Some of the blends in Table 2 are gels with a very attractive set of properties such as: being transparent, self-healing, stretchable, tough, and adhesive. These properties are of great interest for their practical use as battery electrolytes because they point toward good wettability of the electrodes and robustness of the electrolyte. These properties are illustrated in Figure 1 for P50-5/ULi, P50-5/EGLi, and P50-7.5/Pyr13Li2 and in the Supporting Information video for gel P50-5/ULi, which sticks back after breaking and can be immediately stretched again.

The ability of PEO to complex Li^+^ is well-known. Therefore, the dissolution of this polymer in Li-doped electrolytes brings about many effects: it may contribute to the salt dissolution (particularly in the RTIL blends), it modifies the HBD/HBA balance in the DES electrolytes, and it plays a very significant role in the rheology of these gels because of the physical cross-linking produced by the Li/PEO interchain interaction.^25^ Thus, the molar ratio Li^+^/EO is a very relevant aspect in these blends, and because of that it is included in Table 2.

### Effect of the Composition and PEO Chain Length on the Rheology of DES and RTIL Gels

The rheology of the blends is studied at 25 and 75 °C because electrolytes in batteries may need to endure temperatures up to 70 °C, where PEO melts. Ion conductivity (σ) has been measured in the range −50 to 90 °C. Table 2 collects the crossover frequency, *G*′ = *G*″ at 25 and 75 °C, the elastic modulus at 100 rad s^–1^, *G*′_100_, and σ at 25 °C.

**Figure 2:**
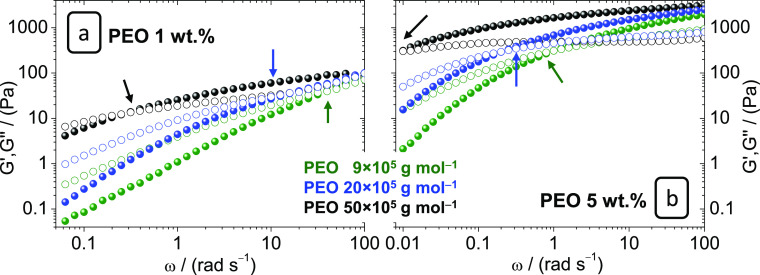
Effect of PEO MW on the rheology of ULi gel electrolytes at 75 °C in the frequency range 0.06–100 rad s^–1^ for gels with 1 wt % of PEO, P9-1/ULi, P20-1/ULi, and P50-1/ULi (a) and 0.01–100 rad s^–1^ for the gels with 5 wt % of PEO, P9-5/ULi, P20-5/ULi, and P50-5/ULi (b). Arrows indicate the crossover frequencies. Solid symbols *G*′ and open symbols *G*″.

The rheological curves of the ULi gels prepared with 1 and 5 wt % at 75 °C are shown in Figure 2a,b, respectively. The rheology data at 25 °C are shown in Figure S2. These gels present the four characteristic rheological regions of polymer materials, which depend on the measurement time. These are, as a function of increasing time: glassy state, transition regime, rubbery state, and terminal stage. This last one is being dominated by viscous and not elastic behavior, that is, liquid-like. The transition from a rubber to a liquid is marked by the crossover frequency, at which the viscous shear modulus *G*″ (loss modulus) becomes equal to the elastic shear modulus *G*′ (storage modulus), being its inverse the network relaxation time.

Figure 2 and Table 2 show how the crossover frequency at 25 and 75 °C shifts toward lower frequencies as the PEO MW and content increase. P1-1/ULi and P1-5/ULi blends have crossover frequencies > 100 rad s^–1^ at 25 and 75 °C and can be considered as liquids. In contrast, P50-5/ULi and P50-2.5/ULi present a solid-like behavior within all the measured frequency range and up to at least 75 °C, with *G*″ = *G*′ lower than 0.01 rad s^–1^.

**Figure 3:**
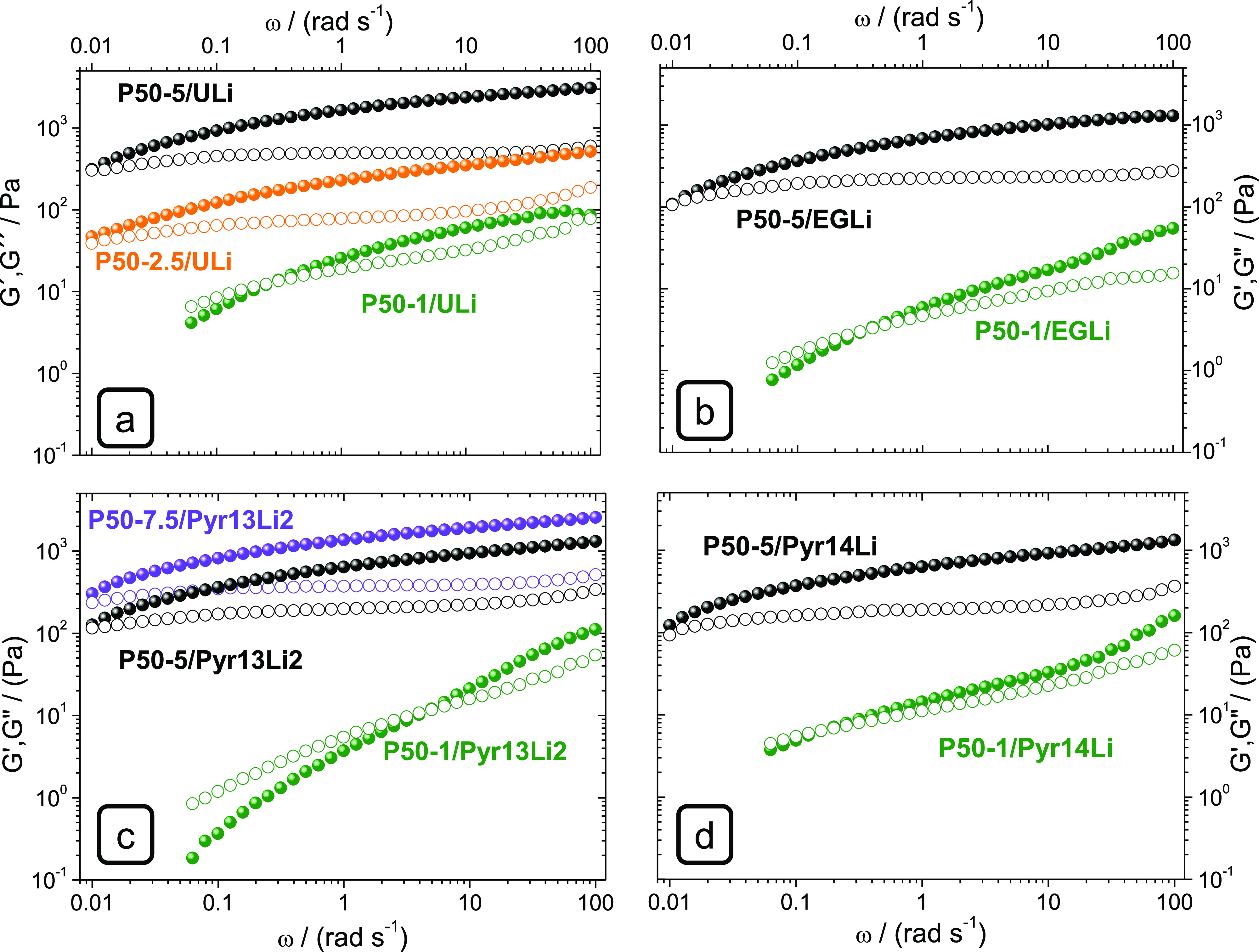
Effect of the PEO (MW = 50 × 10^5^ g mol^–1^) concentration on the rheology at 75 °C in the frequency range 0.06–100 rad s^–1^ of the gel electrolytes ULi (a), EGLi (b), Pyr13Li2 (c), and Pyr14Li2 (d). Solid symbols *G*′ and open symbols *G*″.

According to Figure 2 and Table 2, the rheology of these blends is mainly driven by the polymer chain length rather than the polymer concentration. As an example, notice how the crossover of P9-5/ULi is at higher frequencies than that of P50-1/ULi, that is, creep times of P50-1/ULi will be significantly longer than those of P9-5/ULi. On its turn, the elastic modulus markedly depends on the PEO concentration. This effect is better seen in Figure 3, where the effect of PEO 50 × 10^5^ g mol^–1^ concentration on rheology curves is represented for the different liquid electrolyte systems at 75 °C.

**Figure 4:**
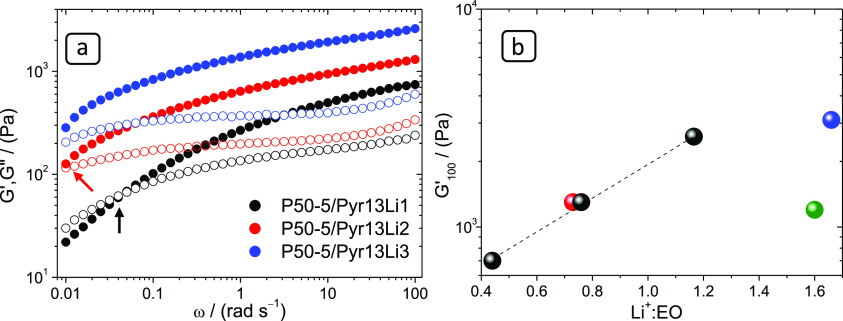
Rheology curves at 75 °C of Pyr13Li 5 wt % gels with different LiTFSI concentrations. G′ solid symbols, G′′ open symbols (a). Log *G*′ (100 rad s^–1^) at 75 °C as a function of Li^+^/EO for the gels prepared with 5 wt % of 50 × 10^5^ g mol^–1^ PEO and Pyr13Li solutions (black), Pyr14Li solution (red), ULi (blue), and EGLi (green) (b).

In Figure 3a, P50-1/ULi, P50-2.5/ULi, and P50-5/ULi gels are compared. The elastic moduli increase by over an order of magnitude when the PEO concentration increases from 1 to 5 wt %. Elastic moduli also increase with polymer concentration in the gels prepared with the other liquid electrolytes, as shown in Figure 3b–d, where the rheological curves of the P50-*n*/EGLi, P50-*n*/Pyr13Li2, and P50-*n*/Pyr14Li2 gels are represented. Comparison of Figure 3a,b shows that the elastic modulus of the EGLi gels is lower than those of analogous ULi ones. However, the crossover of EGLi and ULi with the same PEO concentration is very similar. On their turn, P50-*n*/Pyr13Li2 and P50-*n*/Pyr14Li2 gels show elastic moduli, which are between those of P50-*n*/ULi and P50-*n*/EGLi, where the effect of the increase in the PEO concentration on elastic moduli is also well seen. Together with the PEO MW and concentration, there is a third factor ruling rheology in these gels, which is the Li/PEO ratio because of the PEO cross-linking effect of Li^+^. Eutectic systems are not adequate for the study of the composition effect on properties because their formulation admits little modification. To study these differences, PYR13-TFSI and PYR14-TFSI solutions with different LiTFSI concentrations (Table 2) have been prepared and compared in Figure 4. Figure 4a shows the effect of the Li concentration on PEO/Pyr13Li gels prepared with 5 wt % PEO of MW = 50 × 10^5^ g mol^–1^: it can be observed that the higher the Li concentration, the higher the modulus and relaxation time (crossover occurs at lower frequencies). Figure 4b illustrates the effect of Li^+^/EO on the *G*′ values for the gels in Table 3. All the RTIL electrolytes (P50-5/Pyr13Li1, P50-5/Pyr13Li2, P50-5/Pyr13Li3, and P50-5/Pyr14Li2) fall on the same logarithmic trend, where G′ increases exponentially with Li^+^/EO. While this result was expected, it has an important implication in the context of this work because it shows that in the PyrLi solutions, part of the Li^+^ is complexed at the PEO chains, cross-linking them, thus the concentration of available Li in the liquid phase may decrease on adding PEO to form the gel. As will be shown in a forthcoming section, this has consequences on the ion mobility of these electrolytes.

**Table 3:** PGSE-NMR Diffusion Coefficients, *D*_Li_ and *D*_TFSI_, Li Transport Number, *t*^+^, Nernst–Einstein Ion Conductivity, σ_N_, and  at 25 °C

electrolyte	*D*_Li_ × 10^12^, m^2^ s^–1^	*D*_TFSI_ × 10^1^, m^2^ s^–1^	*t*^+^	σ_N_ × 10^3^, S cm^–1^	α
ULi	1.9	1.5	0.56	0.33	0.88
P1-1/ULi	1.8	1.6	0.53	0.34	0.84
P50-1/ULi	3.3	3.0	0.52	0.62	0.63
EGLi	20.0	14.9	0.57	3.01	0.89
P1-1/EGLi	18.4	12.9	0.59	2.67	0.93
P50-1/EGLi	21.0	15.6	0.57	3.15	0.98
Pyr14Li2	2.3	2.5	0.31/0.48^a^	0.68	0.54
P1-1/Pyr14Li2	2.1	2.0	0.34/0.51^a^	0.55	0.69
P50-1/Pyr14Li2	4.4	4.3	0.34/0.51^a^	1.18	0.58

a , to allow comparison with the EGLi and ULi series.

Figure 4b shows that neither P50-5/ULi (blue) nor P50-5/EGLi (green) fall in the same trend as the PyrLi gels. The amount of well-dissolved Li is much higher in the DES than in the PyrLi electrolytes and therefore the rheology comparison between DES and PyrTFSI gels in the same Li/EO range cannot be made.

The FTIR spectra in Figure S3 show the δ(CF_3_) TFSI band and its modification on adding PEO in Pyr13Li gels and in DES gels. The shift of δ(CF_3_) illustrates how PEO contributes to the LiTFSI dissolution in Pyr13Li gels by complexing Li. On its turn, Li complexed at the PEO chain contributes to the rheology of the gels by cross-linking the polymer chains, as shown in Figure 4. In the case of the DES gels (Figure S3b), this TFSI band is not sensitive to the PEO concentration. This agrees well with the conclusions drawn from the DFT simulations reported in the literature^17,18^ on eutectic mixtures of urea and LiTFSI. These studies show that the diffusivity of all species in ULi DES increases linearly with increasing urea mole fraction, with *D*_Li_ becoming higher than *D*_TFSI_ over a given composition, as occurs in anion exchange Li transport. This peculiar behavior is explained by a successive replacement of TFSI ions by urea^18^ from the first coordination shell around Li ions. The ULi eutectic mixture used in this work is the one with the highest diffusivity values, where all TFSI anions have been replaced by urea molecules in the first coordination shell, with TFSI being at the second coordination shell. Thus, in ULi, TFSI is already weakly coordinated to Li and so it is little sensitive to the addition of PEO. However, the crossover values of DES gels make polymer cross-linking by Li strongly likely also in ULi and EGLi gels.

In summary, the rheology of the gels shows the expected and conventional dependence on the polymer MW and concentration along with on the Li concentration. Gels prepared with 1 wt % UHMW PEO are remarkable because they display long creep times in comparison with those obtained in gels of similar concentrations prepared with polymers, which do not interact with the liquid electrolyte.^12,13^ The longer creep times in these PEO gels are a consequence of the well-known Li^+^ physical cross-linking of PEO chains.

### Effects of the Composition and PEO Chain Length on the Ion Mobility in DES and RTIL Gels

The σ values of the electrolytes at 25 °C are listed in Table 2. The σ values of the pure liquid electrolytes at 25 °C are all in very good agreement with those found in the literature. σ of ULi is reported^16^ to be 0.23 × 10^–3^ S cm^–1^, extrapolation of EGLi to 25 °C from the data in the literature^19^ would be 1.4 × 10^–3^ S cm^–1^, and that of Pyr14Li with a very similar LiTFSI concentration^24^ is 0.4 × 10^–3^ S cm^–1^.

Strikingly, at 25 °C all the gels prepared with 1 wt % PEO have σ values that are the same or higher than those of the pure liquid electrolyte. The effect is more notorious in the PyrLi electrolytes, where the P50-1 gels roughly double the σ values of the pure liquid at 25 °C (see P50-1/Pyr14Li2, P50-1/Pyr13Li1, and P50-1/Pyr13Li2). The effect is also clear in the ULi gels, with P50-1/ULi being about 50% over ULi. σ of P50-1/EGLi is only slightly higher than that of EGLi.

Many of the gels with higher PEO concentrations (2.5 and 5 wt %) also show σ values higher than or equal to those of liquid electrolytes. For instance, σ of Pyr14Li2 at 25 °C (0.37 × 10^–3^ S cm^–1^) is about half that of P50-5/Pyr14Li prepared either manually (this work, 0.86 × 10^–3^ S cm^–1^) or by extrusion^23^ (0.78 × 10^–3^ S cm^–1^). P50-5/Pyr13Li gels display higher σ than Pyr13Li. The ULi gels prepared with 5 wt % PEO of lower MW (P1-5/ULi and P9-5/ULi) also have σ higher than ULi.

**Figure 5:**
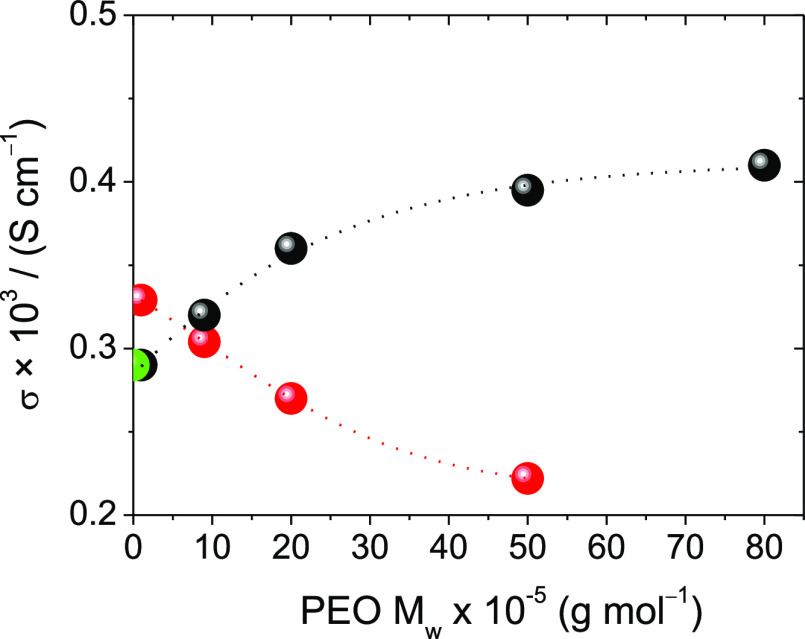
σ (25 °C) in Pm-1/ULi (black) and Pm-5/ULi (red) electrolytes as a function of PEO MW. Pure ULi values are represented in green. Lines are only for guidance.

In fact, this peculiar behavior of the conductivity of the gels is not only concentration but also polymer chain length dependent. See in Table 2 how σ values of P50-1/ULi, P50-1/EGLi, and P50-1/Pyr14Li2 are higher than those of the gels prepared with the lower MW PEO P1-1/ULi, P1-1/EGLi, and P1-1/Pyr14Li2. Figure 5 shows σ at 25 °C of the ULi gels as a function of PEO MW for 1 and 5 wt % gels. In the electrolytes of the Pm-1/ULi series (in black), σ increases with the MW of PEO, in such a way that σ of P50-1/ULi is about 50% higher than σ of pure ULi.

The more concentrated Pm-5/ULi electrolytes (in red) show a negative dependence of σ on PEO MW, which is the conventional behavior in dissolutions of polymers of increasing MW. Although it is very remarkable than for the lower MW PEO gels P1-5/ULi and P9-5/ULi, σ is still higher than that of the pure ULi.

**Figure 6:**
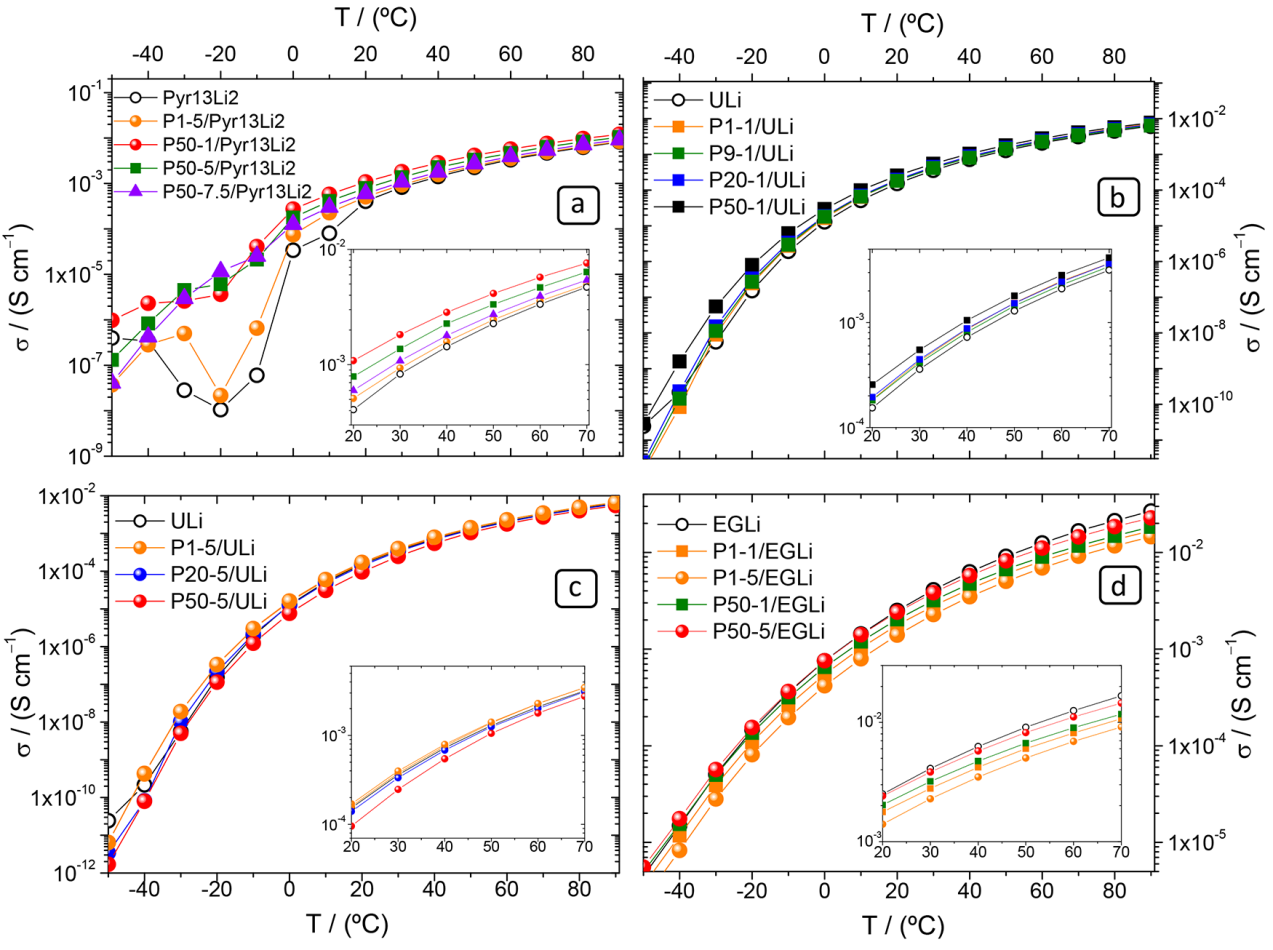
σ in the *T* range −50 to 70 °C of Pyr13Li2 gels (a), 1 wt % ULi gels (b), 5 wt % ULi gels (c), and EGLi gels (d).

A more complete view of the behavior of these blends is found in Figure 6, which collects σ(T) between −40 and 90 °C for ULi and Pyr13Li2 in Table 2. The insets collect σ(T) between 20 and 80 °C. Pyr13Li2 in Figure 6a shows a phase transition at −20 °C. This phase transition is less conspicuous in the gels, and it is almost suppressed in the P50-7.5/Pyr13Li2. The suppression of the phase transition depends more on the PEO MW than on its concentration (compare P1-5/Pyr13Li2 and P50-1/Pyr13Li2), and this is so because of the higher viscosity imparted by UHMW polymer chains. The suppression of the phase transition of Pyr14Li2 in the gel P50-1/Pyr14Li2 can be seen in Figure S1. Although at low *T* (<25 °C), the phase transition suppression can be the reason why the gels prepared with high MW PEO have higher σ than the pure liquid electrolytes, it does not explain why they continue to be higher up to 90 °C.

Figure 6b shows that in the Pm-1/ULi system, all blends have higher σ than ULi, not only at 25 °C, but throughout the full T range. The highest difference is for the higher MW PEO (20 × 10^5^ and 50 × 10^5^ g mol^–1^). In the more concentrated Pm-5/ULi system (Figure 6c), only P1-5/ULi and P9-5/ULi (not shown in the figure for the sake of clarity) have higher σ than ULi in all the *T* range. In the EGLi series, as *T* increases, σ of pure EGLi becomes progressively higher that of the gels.

In this connection, it is very interesting that no phase transitions are detected in the σ(*T*) representation of ULi gels or the EGLi ones. Because PEO is a Lewis base (like urea and EG), it is very reasonable to assume that it will participate to a certain extent as HBD in the eutectic mixtures EGLi and ULi. Then, the HBD/HBA ratio in ULi and EGLi in the gels is higher than that in the pure DES. If HBD in these systems is taken as HBD_PEO_ + HBD_urea_ (or HBD_PEO_ + HBD_EG_), then HBD/HBA ratios in 1 wt % gels are 3.6:1 and 4.2:1 and in 5 wt % gels 4.1:1 and 4.8:1 in ULi in EGLi, respectively. According to the literature, in ULi the ratio 3.6:1 can still be considered to be at the eutectic point, but the ratio 4.1:1 is clearly away from it, and a solid phase melting close to 50 °C coexists with the phase, which melts at −50 °C. However, Figure 6b,c shows no phase transitions in the T range −50 to 90 °C. This means that, either the addition of PEO at those concentrations is not significantly shifting the HBD/HBA ratio of ULi away from its eutectic point or that, as in the case of the Pyr13Li2 gels in Figure 6a, the polymeric nature of the PEO hinders the phase separation into liquid-rich and solid-rich phases.

That PEO behaving as an HBD in the EGLi and ULi gels is extremely likely because of the strong interaction of Li with PEO and EG. Moreover, in the particular case of ULi, PEO and urea are known to interact very strongly, even forming inclusion compounds (ICs).^26^ The hypothesis we propose is that in the DES PEO gels, the phase separation that could arise from the HBD/HBA ratio increase is hindered by the presence of the polymer. In any case, in the DES gels the HBD/HBA ratio is different from that of the pure liquids, and this has to be borne in mind when comparing the σ values of the pure DES and their gels.

In PyrLi gels, the addition of PEO also changes the chemical structure of the liquid by contributing to the better dissolution of LiTFSI, as shown in Figure S3. In a previous work,^21^ we described how the addition of short-chain polyethylene glycol (PEG 550 g mol^–1^) to solutions of Li-doped ionic liquids strongly reduces their viscosity (η). For instance, the ternary dissolution of PEG, LiTFSI, and PYR13-TFSI has a η value, which is 4-fold lower than that of the dissolution of LiTFSI in PYR13-TFSI. This occurs because PEG chains are able to complex Li cations. In consequence, Li coordination is shared between the EO units of PEG and the TFSI anions, which makes this blend less viscous than the dissolution of LiTFSI in PYR13-LiTFSI. In all the PyrLi-based electrolytes in Table 2, PEO complexes Li cations, decreasing in this way the interaction between Li and TFSI in the liquid phase. The σ enhancement in the PyrLi blends with higher PEO concentrations (P1-5/Pyr13Li2 and P1-5/Pyr14Li2) with respect to either the liquid electrolyte or the P1-1/x blends can be understood in the same way. Further addition of PEO promotes more Li cations being complexed by EO units, further releasing TFSI from its interaction with Li. This effect on the PyrLi electrolyte chemical structure is concentration-dependent, very much like the HBD/HBA ratio modification in the DES.

For a better understanding of ion mobility in these gels at a molecular level, the diffusion coefficients, *D*_Li_ and *D*_TFSI_, are measured in some selected electrolytes and are shown in Table 3. Ion diffusivity is about 2-fold higher in P50-1/ULi and P50-1/Pyr14Li2 gels than that in ULi and Pyr14Li2 or in the P1-1/ULi and P1-1/Pyr14Li2 gels. Then, the remarkable increase in the σ of the ULi and PyrLi P50-1 gel electrolytes has a correlation at the molecular scale, and diffusivity in these gels is clearly higher than diffusivity in the liquid electrolyte or the gels prepared with the lower MW PEO. In the case of the EGLi system, *D* values are roughly the same in the liquid and the P50-1/EGLi gel.

Table 3 also includes the Li transport number, *t*^+^, calculated using eqs 1 and 2. The latter was used for the Pyr14Li2 electrolytes, where *D*_PYR_ was considered equal to *D*_TFSI_.^27^
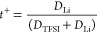
1
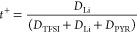
2*t*^+^ offers information on the diffusion mechanisms: when transport is viscosity (η) governed, large entities diffuse more slowly because, according to the Stokes–Einstein equation, *D* is inversely proportional to the hydrodynamic radius of the diffusing ion. Li, which has a large solvation shell, diffuses more slowly than TFSI in η-governed electrolytes. When Li moves via other transport mechanisms, for instance anion exchange, the diffusivity of Li can increase over that of TFSI. This is the case for ULi and EGLi, where *t*^+^ is over 0.5.

Table 3 shows how *t*^+^ does not change either in the EGLi or the Pyr14Li systems, implying that the addition of PEO does not substantially change their transport mechanism. According to the DFT simulations, the theoretical value of *t*^+^ in ULi 3.5:1 is 0.58, which is in good agreement with the experimental value of *t*^+^ = 0.56^18^ found in this work. The addition of only 1 wt % of PEO decreases *t*^+^ from 0.56 to 0.52, suggesting that in this system the transport mechanism may become more η-governed because of the presence of the polymer.

Table 3 includes the Nernst–Einstein conductivity σ_N_, obtained using eq 3
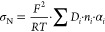
3where *F* is the Faraday constant and *D*_*i*_, *n*_*i*_, and α_*i*_ are the diffusion coefficients, the concentrations, and the ionicities of the *i* species. σ_N_ in Table 3 is calculated assuming that α_*i*_ = 1, that is, dissociation of the ionic species is complete. Then, the ratio  in Table 3 is a measure of how true this assumption is: the lower α, the less dissociated the ionic species are. For instance, the presence of aggregates or ion pairs instead of free ions decreases α.

Interestingly, in the Pyr14Li2 and the EGLi blends prepared with the lower MW PEO P1-1 (which are not gels but liquids according to rheology), diffusivity is slightly lower than in the liquid. However, see in Table 2 that σ values of P1-1/EGLi and P1-1/Pyr14Li2 are not below the σ value of EGLi or Pyr14Li2, respectively. Table 3 shows that, in these two systems, the addition of PEO (irrespective of its chain length) brings about an increase in ionicity α, meaning that a higher concentration of free ions is able to contribute to σ. In the Pyr14Li series, this is caused by the more complete dissolution of the LiTFSI salt thanks to the Li complexation by PEO, as explained before. The α increase in EGLi gels is not accompanied by modifications of *t*^+^. Therefore, apparently, the addition of PEO does not change the diffusion mechanism, which suggests that a small concentration of PEO may be simply fine-tuning this DES formulation.

To explain the ion mobility (σ and D) enhancement and the *t*^+^ decrease found in the ULi gels, we propose that (i) PEO interacts strongly with urea and (ii) it substitutes urea from the Li coordination shell. On the one hand, the interaction of PEO with urea will fix (to a certain extent) the positions of the urea coordination shells of Li along the PEO chain and will loosen the interaction of Li with urea. Both effects can yield higher diffusivity for Li (and also for the TFSI anions at the second coordination shell). The FTIR spectra of ULi gels and urea/PEO IC in the 700–1000 cm^–1^ region, which are shown in Figure S4, suggest that the interaction of urea with PEO does occur. On the other hand, it is very strongly suggested by the rheology of the ULi gels that PEO complexes Li, substituting urea in the first coordination shell of the cation. This Li–PEO interaction will promote the anion exchange transport of Li along the polymer chain and/or the appearance of the conventional Li transport along the PEO chain, which depends on the PEO chain segmental motions.

**Scheme 2:**
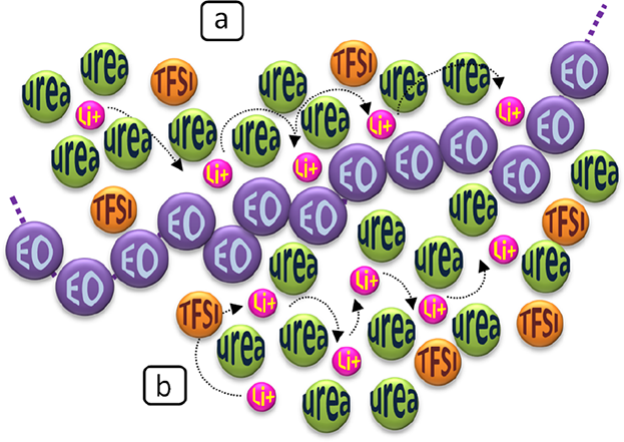
Proposed Structure for ULi Gels, Showing the Direct Interaction of EO with Li (a) and Urea (b), and Their Effect on Li Transport

Scheme 2 illustrates these mechanisms. Either by complexing Li as shown in Scheme 2a or by interacting with urea as shown in Scheme 2b, the polymer chains will foster the liquid-phase organization, which can lead to higher ion mobility. Because of the polymer chain involvement in the ion transport, it may become somewhat viscosity dependent, explaining the *t*^+^ decrease in the ULi gels. In PyrLi and EGLi gels, the participation of PEO by complexing Li, as in Scheme 2a, can also be taking place.

**Figure 7:**
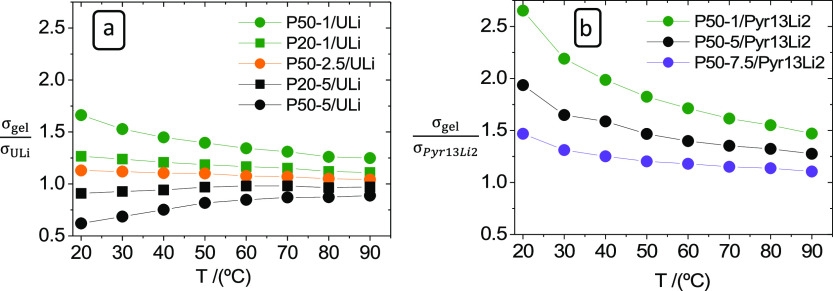
Ratio of  (a) and  (b) as a function of temperature for gel electrolytes prepared with PEO of different MWs and concentrations.

The mechanisms proposed in Scheme 2 for ULi gels or the modifications in the chemical structure of the PyrLi gels described above can explain that the presence of PEO increases diffusivity, but on their own they do not explain the chain length dependence of this phenomenon. This chain-length dependent increase of diffusivity can be understood in the light of Archer’s findings^12,13^ on the uncoupling of rheology and ion mobility in elastic gel networks prepared with very low concentrations of UHMW polymers. According to this, in gels where the uncoupling occurs, the ion mobility is that of the liquid phase. The effect of the formation of the elastic polymer gel network on σ of ULi gels is illustrated in Figure 7, where the ratios  and  are represented in the range 10 to 90 °C. In all the T range studied, P50-1/ULi, P20-1/ULi, and P50-2.5/ULi have  values over 1. Likewise,  is over 1 for P50-1/Pyr13Li2, P50-5/Pyr13Li2, and P50-7.5/Pyr13Li2. This σ_gel_ enhancement becomes lower as T increases, strongly suggesting that this phenomenon is connected to the elasticity of the network. In gels that behave conventionally, that is, those with σ lower than that of the liquid, P20-5/ULi and P50-5/ULi in Figure 7,  has a positive temperature dependence because of the polymer viscosity decrease in this temperature range.

**Scheme 3:**
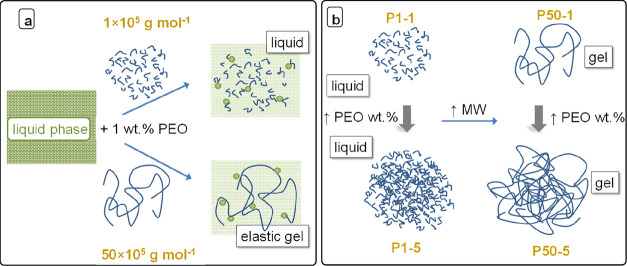
Effect of Adding PEO of Different MWs to the Liquid Electrolyte on (a) Chemical Structure of the Liquid Phase and (b) Viscoelasticity and Mesh Size in the Blend

Scheme 3 summarizes the effect of polymer MW and concentration on σ when a polymer that readily interacts with the liquid electrolyte is chosen to prepare the gel. As discussed in this work, the interaction between the polymer and the liquid electrolyte changes the chemical structure of the liquid phase, and this phenomenon is only concentration-dependent. This is illustrated in Scheme 3a. The change in the chemical structure can lead to a liquid phase with higher ion mobility, as in the PyrLi and ULi series. The modification of the liquid electrolyte chemical structure is only concentration-dependent, but the viscoelasticity imparted by the addition of polymer chains is very strongly chain-length dependent, as illustrated in Scheme 3b.

In the framework of Scheme 3, the lower conductivity and diffusivity of the P1-1 blends in comparison to those of analogous P50-1 gels would be a consequence of the viscous contribution of the shorter PEO chains, which are not sufficiently long as to produce an elastic network.

Though the rheology and ion mobility uncoupling in well-developed gels explain the ion mobility enhancement, it cannot be ruled out that the percolation of elastic PEO chains in the gels (P50-1), which does not occur in liquids with the same concentration (P1-1), may produce a fast and continuous lane for ion diffusion, contributing to an increased ion mobility in ULi, PyrLi, and also EGLi gels.

In summary, if the gel formulation is well chosen, it is possible to prepare viscoelastic gels with σ over that of the liquid electrolyte. These gels are really attractive because of the combination of properties they present, which has been described along this work. It is important to bear in mind that, according to Archer and collaborators, gels in which ion mobility uncouples from rheology are the gels in which electroconvection is reduced during the charging of a battery, where consequently dendritic growth at the anode is eliminated or mitigated. This adds on interest to these gel families as electrolytes in efficient and safer batteries.

## Conclusions

Gels of PEO with Li-doped PYR ionic liquids and the DES urea/LiTFSI and EG/LiTFSI have been prepared using a simple and fast procedure consisting of the polymer dissolution over its melting point in the different liquid electrolytes selected. The rheology and ionic conductivity of the gel electrolytes have been studied as a function of PEO MW and concentration and nature of the liquid electrolyte. Gels prepared with PEO MW > 9 × 10^5^ g mol^–1^ are stretchable, tough, self-healing, sticky, and transparent, and their rheology can be easily tuned by varying the concentration and MW of PEO, making them flow by increasing temperature. At room temperature, many of the gels have ionic conductivity higher than or equal to that of pure liquid electrolytes. The ionic conductivity enhancement is found to depend on the PEO concentration and its MW, in such a way that conductivity enhancement is the maximum in gels prepared with 1 wt % of UHMW PEO. PGSE NMR studies show that the diffusion coefficients of the ions are also higher in the gels prepared with 1 wt % of 50 × 10^5^ g mol^–1^ PEO than in the gels prepared with 1 wt % of 1 × 10^5^ g mol^–1^ PEO or in the pure liquid electrolytes. This unprecedented molecular weight dependence of conductivity and diffusivity results from the modification of the chemical structure of the liquid electrolyte as a consequence of the addition of PEO, and the development of elastic networks, where ion mobility and rheology are uncoupled when UHMW PEO is added. Some of these gels present a very attractive combination of properties because they display liquid-like conductivities together with viscoelasticity, transparency, toughness, and self-healing ability. They have adhesive properties and thus able to wet solid surfaces very well. All these properties make them very appealing for their practical application in multiple energy storage devices.

## Supporting Information Available

Stretching after sticking P50-5/ULi (AVI)
Ion conductivity as a function of temperature of Pyr14Li2 gels; rheology of the ULi and EGLi blends at 25 °C; and FTIR spectra of the liquids and gels (PDF)

## Supplementary Material

ap2c00104_si_001.avi
