# Supplementary material for: Ionic Conductivity Enhancement in UHMW PEO Gel Electrolytes Based on Room-Temperature Ionic Liquids and Deep Eutectic Solvents
Source: ACS Appl Polym Mater. 2022 Mar 25;4(4):2860–70. doi: 10.1021/acsapm.2c00104 (PMC9004316; doi:10.1021/acsapm.2c00104)
Supplement: Supplementary file 2 — ap2c00104_si_002.pdf [file ap2c00104_si_002.pdf]

SUPPLEMENTARY INFORMATION

Ionic conductivity enhancement in UHMW PEO gel electrolytes based on room temperature ionic liquids and deep eutectic solvents

*Víctor Gregorio, Nuria García, Pilar Tiemblo**

ptiemblo@ictp.csic.es

Instituto de Ciencia y Tecnología de Polímeros, ICTP-CSIC, Juan de la Cierva 3, 28006 Madrid, Spain.

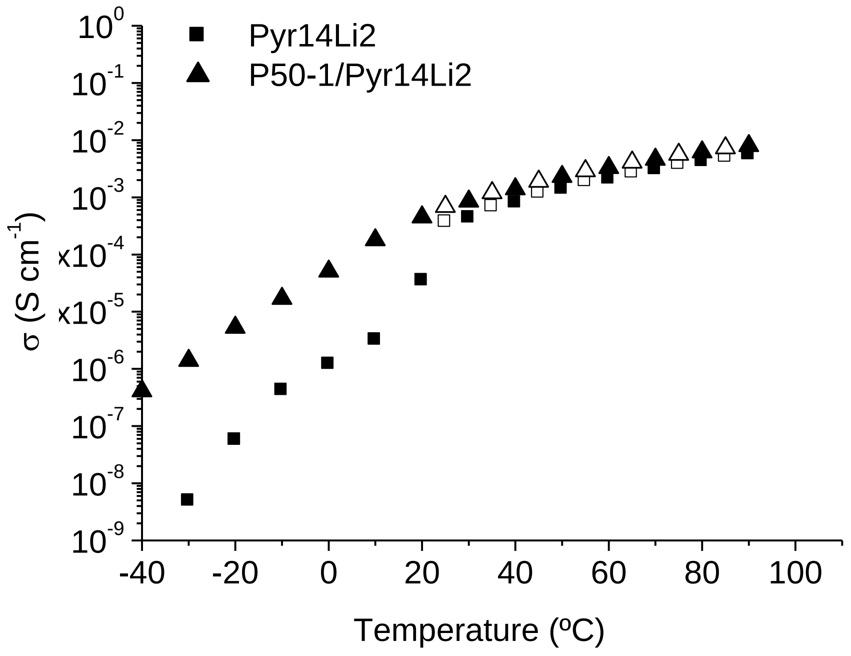

Figure S1: σ(T) on heating (solid symbols) and on cooling (open symbols) for pure Pyr14Li2 and its gel with 1 wt% of PEO Mw= 50 × 10^5 g mol^{-1}, P50-1/Pyr14Li2

The figure shows the phase transition in Pyr14Li2, typical of highly concentrated solutions of LiTFSI in Pyr14TFSI[1]. This phase transition is suppressed in the P50-1/Pyr14Li2 gel. P50-1/Pyr14Li2 is over Pyr14Li2 in all the T range.

Cooling and heating experiments are in the same σ(T) line in the range where they can be compared (25 to 90ºC).

[1] W. A. Henderson, S. Passerini, Phase Behavior of Ionic Liquid−LiX Mixtures: Pyrrolidinium Cations and TFSI- Anions, *Chem. Mater. 2004, 16, 15, 2881–2885*

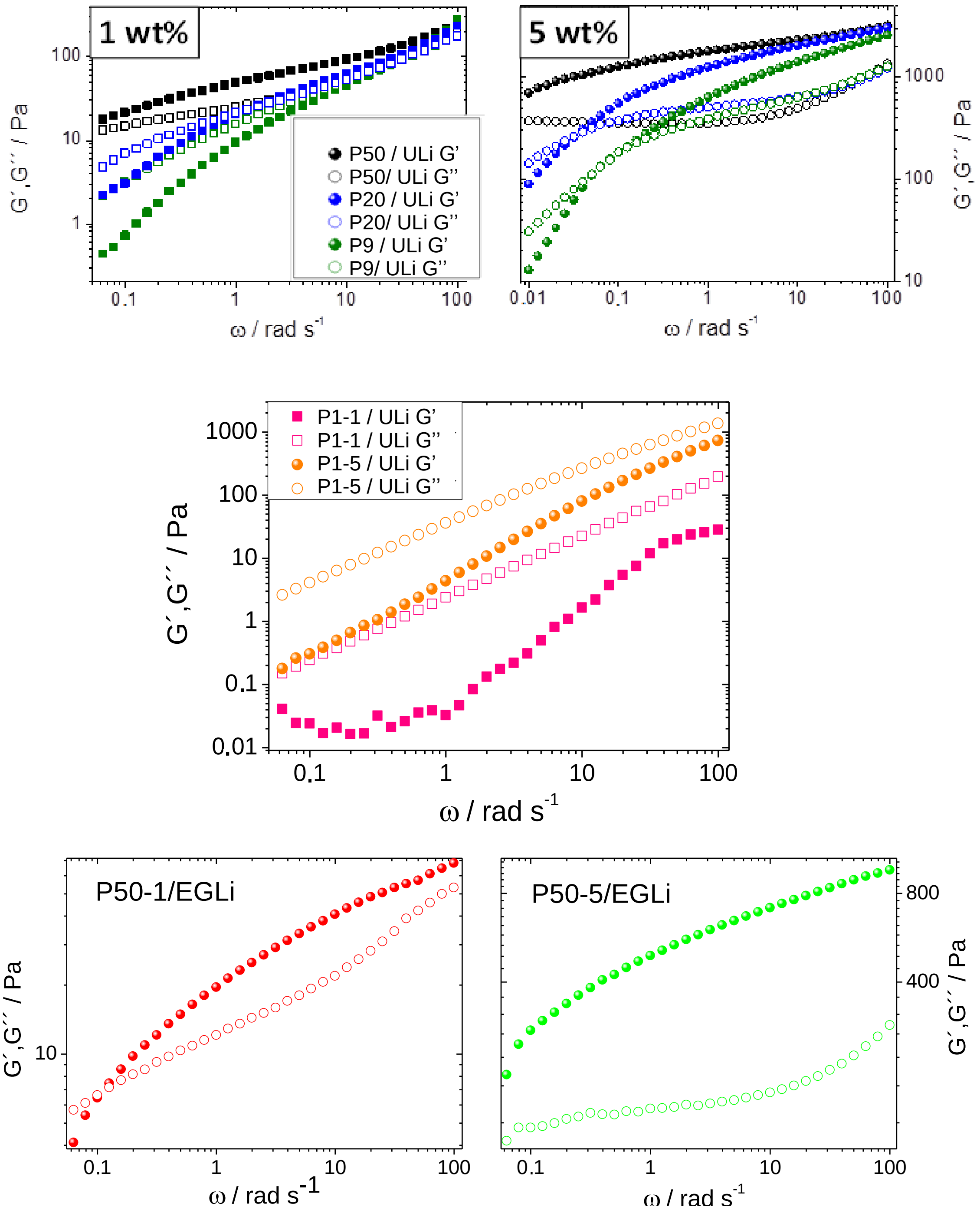

Figure S2. Rheology of the ULi and EGLi blends at 25 ºC.

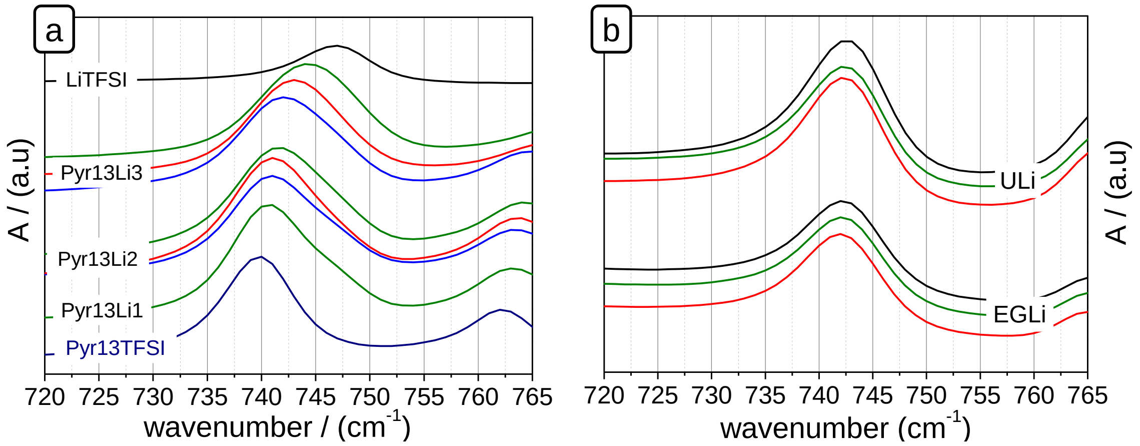

FigureS3: FTIR δ(CF_3) TFSI band in (a) the Pyr13Li gels with PEO 1 wt. % (green), 5 wt. % (red) and 7.5 wt. % (blue) and (b) the DES liquids (black) and their PEO 1 wt. % (green), 5 wt. % (red) gels.

Figure S3 collects the FTIR spectra of the δ(CF_3) TFSI band in Pyr13Li and DES gels. The δ(CF_3) band ranges from 740 cm^{-1} (weakly coordinated TFSI as in Pyr13) and 747 cm^{-1} (strongly coordinated TFSI as in LiTFSI).

Figure S3a illustrates how the TFSI position in Pyr13Li gels depends significantly on the gel formulation, especially on the concentrations of LiTFSI and PEO and their ratio. For instance, in gels with 1 wt% of PEO (Figure S3a, green) and increasing LiTFSI concentration, the band shifts from 741 cm^{-1} in Pyr13Li1 (very close to TFSI in pure Pyr13TFSI) to 745 cm^{-1} in Pyr13Li3 (close to TFSI as in LiTFSI). On the other hand, as the PEO concentration increases (for example in the series P50-1/Pyr13Li2, P50-5/Pyr13Li2 and P50-7.5/Pyr13Li2) the band shifts to positions closer to 740 cm^{-1}, because the complexing of Li by PEO contributes to a better dissolution of LiTFSI. Figure S3b shows that in EGLi and ULi and their gels the band appears at 742 cm^{-1}, as corresponds to a weakly coordinated TFSI, and it is not sensitive to the PEO concentration.

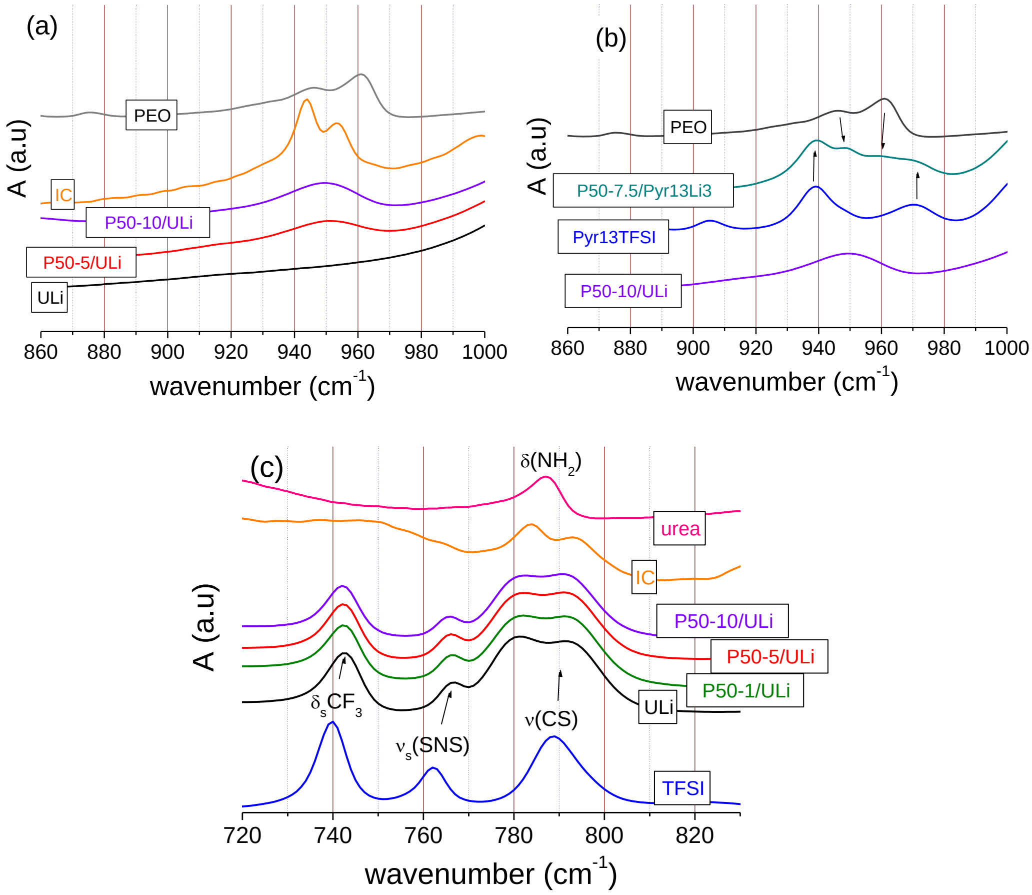

Figure S4: PEO methylene twisting τ(CH_2) in (a) ULi gels and in (b) P50-7.5Pyr13Li3 in comparison with PEO and urea/PEO IC, and (c) δ(NH_2) of urea and υ(CS) of TFSI region between 750 and 800 cm^{-1} in IC and ULi and its gels.

In Figure S4 a and b, τ(CH_2) of pure PEO and its IC with urea is compared with τ(CH_2) in ULi and PyrLi3 gels. The τ(CH_2) in P50-5/ULi and P50-10/ULi is shifted to lower wavelengths as compared to pure PEO, at the same position as in PEO/urea IC. In the P50-7.5/Pyr13Li gel, the envelope of four bands is seen in that region. Two of them correspond well to the Pyr13TFSI spectra, while the other two agree well with the τ(CH_2) positions in PEO. τ(CH_2) suggests that interaction of PEO with urea is taking place.

In Figure S4c, the δ(NH_2) of urea and υ(CS) of TFSI region between 750 and 800 cm^{-1} is shown for urea, the IC and ULi and its gels. In the P50-n/ULi electrolytes the band at 782 cm^{-1} decreases relative to that of 792 cm^{-1} as the concentration of PEO increases. In IC, the δ(NH_2) splits into two bands shifting to higher wavelengths. If in the ULi gels, an interaction between the PEO and the NH_2 of urea is occurring, it could lead to the relative decrease of the 782 cm^{-1} band which occurs on the gels on increasing the PEO concentration.

Though these subtle modifications of the gels FTIR spectra are not a proof of the urea/PEO interaction they do suggest its existence.
